# Supplementary material for: Fusion of 2DGC-MS, HPLC-MS and Sensory Data to Assist Decision-Making in the Marketing of International Monovarietal Chardonnay and Sauvignon Blanc Wines
Source: Foods. 2022 Oct 31;11(21):3458. doi: 10.3390/foods11213458 (PMC9657765; doi:10.3390/foods11213458)
Supplement: Supplementary file 1 [file foods-11-03458-s001.zip › foods-1953717-supplementary/Supporting Information_3 File_revised/Supporting information - File S1_revised.pdf]

Fusion of 2DGC-MS, HPLC-MS, and sensory data to assist decision-making in the marketing of international monovarietal Chardonnay and Sauvignon Blanc wines

Simone Poggesi,[1,2] Aakriti Darnal,[1,2] Adriana Teresa Ceci,[1,2] Edoardo Longo,[1,2,*] Leonardo Vanzo,[1,2] Tanja Mimmo,[2] Emanuele Boselli[1,2]

AFFILIATIONS

[1] Oenolab, NOI Techpark Alto Adige/Südtirol, Via A. Volta 13B, 39100 Bolzano, Italy

[2] Free University of Bozen-Bolzano, Faculty of Science and Technology, Piazza Università 5, 39100 Bolzano, Italy

*corresponding author: edoardo.longo@unibz.it

Supporting Information

Table S1. Target analyte finding parameters.

Compound	Start Time I dim (min)	End Time I dim (min)	Start Time II dim (s)	End Time II dim (s)
butanoic acid, ethyl ester	8.67	12.17	1.45	1.94
hexanoic acid, ethyl ester	17.50	19.30	1.83	2.35
acetic acid, hexyl ester	19.67	21.17	1.80	2.14
4-mercapto-4-methyl-2-pentanone	24.50	25.20	1.19	1.31
2-isopropyl-3-methoxypyrazine	26.80	27.50	1.56	1.83
furfuryl mercaptan	26.80	27.40	1.03	1.19
octanoic acid, ethyl ester	27.00	27.75	2.20	2.60
1-octen-3-ol	27.93	28.53	1.13	1.29
2-sec-buthyl-3-methoxypyrazine	29.70	30.30	1.67	1.88
benzaldehyde	30.62	31.08	1.01	1.21
trans-2-nonenal	31.14	31.74	1.47	1.65
linalool	31.98	32.37	1.25	1.48
decanoic acid, ethyl ester	35.18	35.85	2.30	2.60
α-Terpineol	37.78	38.20	1.18	1.47
3-mercapto-hexyl acetate	38.28	39.00	1.23	1.55
β-Damascenone	42.03	42.33	1.46	1.70
dodecanoic acid, ethyl ester	42.67	43.67	2.45	2.70
3-mercapto-hexanol	43.10	43.40	0.85	1.10
4-ethylguaiacol	49.00	49.50	0.95	1.12
nerolidol	49.63	50.10	1.48	1.68
m-cresol	51.00	51.60	0.76	0.94
ethyl-cynnamate	52.17	52.77	1.07	1.31
methyl anthranilate	54.83	55.30	0.79	1.01
Hexadecanoic acid, ethyl ester	55.25	55.60	1.70	2.00
octadecanoic acid, ethyl ester	58.25	58.67	1.45	1.70

Table S2. Tentative compound identification obtained using Agilent Mass Hunter. The compounds followed by the asterisk were assigned by observing the mass fragmentation, whereas, all the other compounds were identified by injecting the related standard compounds. The data of the fragmentation is reported in the Table S17 & S18 file in the Excel Supporting Information File 2.

Code CH	Code SA	Compound assignement	R.t. (min)	ESI – mass (m/z)	MS² fragments *m/z* (abundance %)	λ max (nm)
x.31 (2.8)	x.20(1.8)	Gallic acid	2.8	169		265
x.68	x.39 (3.8)	Protocatechuic acid	5.5	153	109 (100), 108 (51), 91 (3.5), 65 (1.44)	260
		Hydroxytyrosol*	5.7	153	123 (100), 122 (10)	280
	x.59 (5.2)	cis-caftaric acid*	6.1	311		297, 327
x.96	x.59 (5.2)	trans-caftaric acid	6.9	311	135 (100), 149 (33), 179 (61)	287, 317
x.105		GSP (glutathionyl caftaric acid) *	7.1	616		327, 311
x.118	x.82 (6.3)	cis-cutaric acid*	8.1	295		287, 310
x.124	x.86 (6.6)	trans-cutaric acid *	8.5	295		287, 312
x.133	x.96 (7.2)	(+)-catechin	9.0	289		279
/	x.101 (7.5)	cis-fertaric acid *	9.3	325		285, 315
/	/	trans-caffeic acid	9.5	179		295, 323
x.141		trans-fertaric acid*	9.5	325		297,326
x.164	x.117(8.7)	Epicatechin	10.8	289		273
/	/	*p*-coumaric acid	11.7	163		293, 327
x.180	x.129 (11.3)	Astilbin	14.1	449		291, 335
/	/	Resveratrol	16.7	227		305, 323

Table S3. Single ion monitoring (SIM) acquisition parameters for analysis of PAC in Chardonnay and Sauvignon Blanc wines. Total scan cycle time = 500 ms. (MS² resolution is +/- 0.7 amu)

Compound	Mass (m/z)	MS² Res	Dwell Time (ms)	Identified retention times (+/- 0.1 min)
hexamers (PC)	1731.5	Unit (*)	16	Not observed or below noise level
c-hexamer (PC)	1729.5	Unit	16	Not observed or below noise level
5OH-pentamers (PD)	1523.4	Unit	16	Not observed or below noise level
c-5OH-pentamers (PD)	1521.4	Unit	16	Not observed or below noise level

4OH-pentamers (PD)	1507.4	Unit	16	Not observed or below noise level
c-4OH-pentamers (PD)	1505.4	Unit	16	Not observed or below noise level
3OH-pentamers (PD)	1491.4	Unit	16	Not observed or below noise level
c-3OH-pentamers (PD)	1489.4	Unit	16	Not observed or below noise level
2OH-pentamers (PD)	1475.4	Unit	16	Not observed or below noise level
c-2OH-pentamers (PD)	1473.4	Unit	16	Not observed or below noise level
OH-pentamers (PD)	1459.4	Unit	16	Not observed or below noise level
c-OH-pentamers (PD)	1457.4	Unit	20	34.6
pentamers (PC)	1443.4	Unit	16	34.5 42.0
c-pentamer (PC)	1441.4	Unit	50	24.6
4OH-tetramers (PD)	1219.3	Unit	16	Not observed or below noise level
c-4OH-tetramers (PD)	1217.3	Unit	16	Not observed or below noise level
3OH-tetramers (PD)	1203.3	Unit	50	Not observed or below noise level
c-3OH-tetramers (PD)	1201.3	Unit	16	22.1
2OH-tetramers (PD)	1187.3	Unit	50	Not observed or below noise level
c2OH-tetramers (PD)	1185.3	Unit	50	Not observed or below noise level
OH-tetramers (PD)	1171.3	Unit	50	30.9
c-OH-tetramer (PD)	1169.3	Unit	50	22.2
tetramers (PC)	1155.3	Unit	50	28.1 36.7 37.0
c-tetramer (PC)	1153.3	Unit	50	22.8
Trimers OH (PD)	883.2	Unit	16	18.0
Trimers (PC)	867.2	Unit	16	35.6
Dimers OH2 (PD)	611.1	Unit	16	17.2
Dimers OH (PD)	595.1	Unit	16	27.6 30.9
Dimers (PC)	579.1	Unit	16	29.6 30.9 34.5 37.8

PC = procyanidin
PD = prodelphinidin

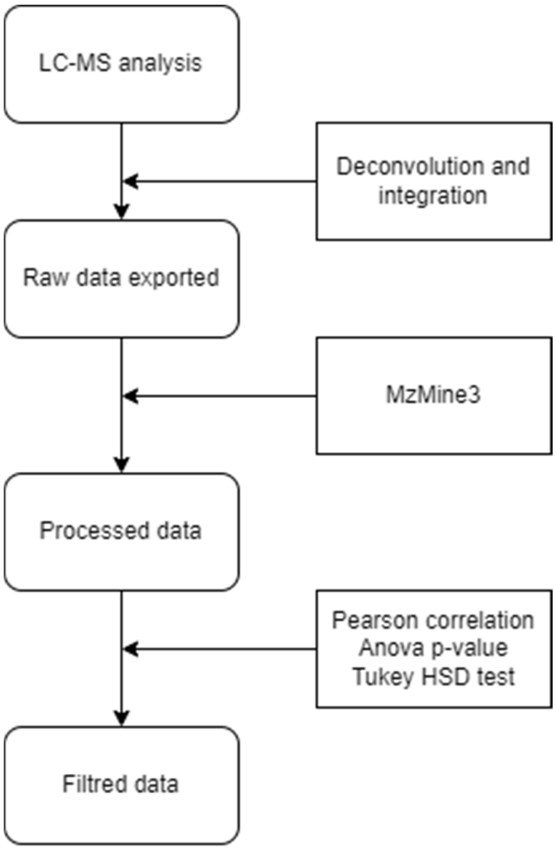

Figure S1. scheme for pre-processing data obtained by LC-MS analysis.

Table S4. LS mean and group (indicated by the letter) obtained by the ANOVA one-way and Tukey HSD. post hoc test for the significant sensory analysis data for the Chardonnay wines (α = 0.05).

	Green colour	Yellow colour	Citrus fruit aroma	Warmness	Tropical fruit flavour	Woody flavour
NZL MA	1.438 ab	1.875 a	2.375 ab	3.000 a	1.875 a	2.500 a
NZL HA	1.688 a	2.250 a	2.125 ab	2.375 ab	2.063 a	2.063 ab
ARG ME	0.875 b	2.875 a	1.438 b	2.813 ab	2.000 a	2.563 a
AUS SE	1.375 ab	1.875 a	1.938 ab	2.688 ab	2.625 a	1.875 abc
RSA CO	1.250 ab	1.563 a	2.688 a	2.688 ab	2.188 a	1.125 cde
RSA WC	1.250 ab	2.625 a	2.563 a	2.938 a	2.000 a	1.438 bcd
CLE CV	1.563 ab	2.438 a	1.938 ab	3.063 a	2.313 a	2.125 ab
ITA SP	1.125 ab	1.500 a	1.500 b	2.188 b	1.813 a	0.625 e
ITA VF	1.125 ab	1.563 a	2.438 ab	2.375 ab	1.813 a	0.813 de
Pr > F(Model)	0.036	0.027	0.009	0.008	0.047	<0.0001

Table S5. LS mean and group (indicated by the letter) obtained by the ANOVA one way and Tukey HSD post hoc test for the significant sensory analysis data for the Sauvignon Blanc wines (α = 0.05).

	Green colour	Yellow colour	Tomato stem aroma	Green pepper aroma	Overall quality judgment
NZL MA	1.000 a	1.000 bc	1.750 a	1.000 ab	3.917 a
ITA VF	0.917 a	2.000 ab	1.000 ab	0.750 ab	3.333 a
RSA WC	1.167 a	1.750 abc	0.667 ab	0.667 ab	3.500 a
CLE CV	0.833 a	1.917 ab	0.667 ab	0.417 ab	3.750 a
AUS SE	0.750 a	1.917 ab	0.750 ab	0.417 ab	3.583 a
ARG ME	0.833 a	2.083 a	0.917 ab	0.417 ab	2.333 b
RSA CO	0.667 a	1.500 abc	2.000 a	1.917 a	3.167 ab
ITA SP	0.417 a	1.500 abc	0.250 b	0.167 b	3.417 a
NZL MR	0.417 a	0.833 c	0.667 ab	0.417 ab	3.417 a
Pr > F(Model)	0.044	0.008	0.015	0.037	0.003

Table S6. LS mean and group (indicated by the letter) obtained by the ANOVA one way and Tukey HSD post hoc test for the significant volatile compounds for the Chardonnay wines ($\alpha = 0.05$).

	I	II	III	IV	V	VI	IX	X	XI	XII	XIII	XIV	XV	XVI	XVIII
NZL HA	10351.952 b	11149625.665 a	20369903.025 a	3837288.176 c	320666.428 b	164883.080 bcd	1497739.657 c	4273838.233 a	12773443.915 a	13526823.020 bc	6479841.333 b	24968459.270 a	20300.059 b	1149321.070 a	329453.290 ab
NZL MA	6012.576 b	566529.184 b	532275.810 b	4459541.671 c	36095.331 b	192574.090 bcd	7287932.188 abc	2096101.194 ab	6366934.629 ab	25918011.090 a	6563551.592 b	347015.844 b	22886.203 b	90104.296 b	261343.044 b
ARG ME	20490.925 b	130399.077 b	345235.803 b	3819302.566 c	63230.559 b	293501.100 abc	3434355.061 bc	2167702.246 ab	4486114.556 ab	23106159.565 a	20441064.620 a	672839.025 b	24934.638 b	166628.640 b	189552.715 b
RSA CO	10613.426 b	182941.024 b	387942.610 b	5298252.375 c	90098.103 b	414320.138 a	7819982.810 abc	624986.478 ab	4636845.814 ab	13608681.875 bc	2604018.692 bc	583913.914 b	59999.740 b	224363.156 b	435087.307 ab
AUS SE	6424.088 b	200876.036 b	352197.096 b	4306010.736 c	33018.897 b	348571.186 ab	4568464.754 abc	1743811.459 ab	4255319.636 b	13642028.210 bc	3537283.600 bc	661998.426 b	618730.566 a	161852.614 b	411593.631 ab
CLE CV	94143.347 a	92329.073 b	270251.295 b	5188715.096 c	29684.454 b	462740.175 a	2209403.489 c	231027.988 b	291162.034 b	20407145.550 a	6142455.108 b	558941.935 b	34088.234 b	203416.142 b	94479.321 b
RSA WC	3951.529 b	176478.179 b	236835.294 b	2833446.442 c	11201.316 b	211759.899 bcd	9007518.041 ab	246880.513 b	5867691.787 ab	12319986.120 c	1794686.459 bc	494073.745 b	10713.395 b	34943.789 b	949757.413 a
ITA VF	9079.741 b	236554.771 b	387383.773 b	13750256.220 b	2616303.985 a	154003.976 cd	11174447.348 a	194395.675 b	1760233.504 b	19821630.715 ab	1777689.041 bc	654774.353 b	11128.050 b	61704.857 b	94181.267 b
ITA SP	7333.221 b	130939.806 b	175826.871 b	17865674.950 a	42057.715 b	102458.035 d	7742318.420 abc	197362.886 b	259036.656 b	13836866.630 bc	656060.888 c	562161.581 b	17796.249 b	127883.343 b	48555.049 b
Pr > F(Model)	<0.0001	0.001	0.001	<0.0001	<0.0001	0.000	0.004	0.026	0.005	0.000	<0.0001	0.001	0.002	0.011	0.007

	XIX	XX	XXI	XXII	XXIII	XXIV	XXV	XXVI	XXVII	XXVIII	XXIX	XXX	XXXI	XXXII	XXXIII	XXXV	XXXVI
NZL HA	67411798.875 a	1456051.022 a	446269.789 b	458626.722 e	2424920.971 a	727895.071 ab	35561.117 b	23057999.520 ab	5118965.720 a	5755233.056 b	13886929.515 a	68445539.360 bc	6623383.248 a	210978706.700 a	16591040.340 a	5644128.321 a	1285929.993 a
NZL MA	48961397.175 ab	1673984.045 a	445151.624 b	1384545.024 cd	2349165.389 a	210375.804 c	33457.109 b	30434475.330 ab	4833696.421 a	8611658.997 ab	9338840.043 ab	224543117.650 a	2959516.800 a	6944499.822 b	635682.752 b	5307884.880 ab	606712.774 b
ARG ME	50786518.735 ab	1897093.537 a	41958.710 b	862614.544 de	3073323.935 a	268404.564 c	28294.909 b	10017463.928 b	4528197.416 a	2514260.623 b	9983278.333 ab	183577248.150 a	3226266.888 a	10138174.873 b	247518.878 b	5663288.892 a	553687.278 b
RSA CO	48366123.340 ab	1677992.336 a	373584.336 b	1790161.050 bc	2201339.555 a	405004.375 bc	24589.223 b	13172579.430 ab	7093413.614 a	19222877.781 ab	9389424.865 ab	60023837.230 bc	877213.817 a	8258124.796 b	275913.010 b	5067371.996 ab	547851.882 b
AUS SE	48023137.400 b	1534286.593 a	392583.957 b	1022255.891 cde	2041877.025 a	370135.854 bc	32603.245 b	42985090.600 a	4640849.191 a	4270113.377 b	8491760.586 ab	75062811.250 bc	2076899.770 a	7294659.628 b	277549.726 b	5422621.799 ab	564751.346 b
CLE CV	42453139.235 b	861859.754 a	99459.547 b	978218.488 cde	924783.445 a	802936.225 a	87006.306 a	13710058.415 ab	8068449.779 a	44535787.140 ab	4410939.416 ab	104628652.115 b	448732.531 a	5071147.464 b	115627.765 b	4921238.919 ab	236512.886 b
RSA WC	53504855.850 ab	880782.989 a	1339690.715 a	2683776.574 a	981842.073 a	195439.929 c	29234.016 b	45232968.005 a	8176489.511 a	40960460.760 ab	4795448.928 ab	30899307.170 c	389653.786 a	5027499.722 b	159321.125 b	4826305.851 ab	726484.659 ab
ITA VF	41342134.015 b	843700.116 a	262412.373 b	2999117.273 a	761902.914 a	231136.357 c	22546.178 b	3940965.171 b	9241502.262 a	49724972.605 a	3024310.101 b	89767100.105 bc	392418.806 a	4667445.922 b	284062.568 b	4412859.055 ab	228203.045 b
ITA SP	38441542.935 b	765679.688 a	17733.318 b	2303083.873 ab	855421.188 a	469897.798 abc	20060.147 b	418318.660 b	8225477.042 a	29301393.315 ab	4210287.097 ab	45403009.285 bc	465732.100 a	4557401.193 b	119653.388 b	3896687.613 b	192672.182 b
Pr > F(Model)	0.007	0.021	0.004	<0.0001	0.023	0.001	<0.0001	0.003	0.016	0.008	0.028	<0.0001	0.091	0.004	0.001	0.035	0.001

Table S7. LS mean and group (indicated by the letter) obtained by the ANOVA one way and Tukey HSD post hoc test for the significant volatile compounds for the Sauvignon Blanc wines (α = 0.05).

	IV
ITA SP	141428.000 a
AUS SE	0.000 b
NZL MR	87312.500 ab
NZL MA	0.000 b
CLE CV	46146.500 ab
ITA VF	0.000 b
RSA WC	41278.000 ab
RSA CO	0.000 b
ARG ME	0.000 b
Pr > F(Model)	0.005

Table S8. LS mean and group (indicated by the letter) obtained by the ANOVA one-way and Tukey HSD post hoc test for the significant phenolic compounds for the Chardonnay wines (α = 0.05).

	x.5	x.6	x.8	x.9	x.11	x.14	x.16	x.17	x.18	x.19	x.20	x.22	x.24	x.26	x.27	x.28	x.29	x.31	x.32
CLE_CV	898.500 b	12117.000 ab	2338.500 abc	5182.000 c	6712.000 bc	2647.000 c	7351.000 b	2529.000 bc	3285.000 a	9330.000 ab	5524.500 ab	8472.000 a	2702.500 b	7303.500 b	2072.000 a	2817.000 bcd	1994.500 c	44517.500 cde	7108.000 a
RSA_CO	1009.500 b	15204.000 ab	1881.500 bcd	3170.500 cd	7527.000 b	1466.500 de	7608.500 b	2786.000 b	2493.000 ab	4125.000 c	4900.500 abcd	4049.500 c	7149.000 a	7983.000 ab	2139.500 a	5571.500 ab	1977.000 c	54931.500 c	5421.000 bcd
RSA_WC	1375.000 a	13029.000 ab	1176.500 d	8071.500 b	9061.000 b	4090.000 b	5792.500 bc	1957.500 bcd	2346.000 ab	9942.000 ab	6075.500 a	5694.500 abc	6532.500 a	9921.500 a	2106.500 a	4677.500 abc	3394.500 b	40797.500 de	6810.000 ab
AUS_SE	1181.000 ab	11332.500 ab	1588.500 cd	4738.500 c	6779.500 b	2629.000 c	6831.500 b	2332.500 bc	2583.000 ab	6471.000 bc	5269.500 abc	4485.500 c	5707.000 a	6343.500 bc	2122.000 a	7691.000 a	2539.500 c	36189.000 ef	6985.000 ab
ITA_VF	1390.500 a	15988.500 a	1946.500 bcd	4809.500 c	6797.000 b	2041.500 cd	11580.000 a	3983.000 a	2772.500 ab	4386.500 c	5762.000 a	5691.500 abc	2260.000 b	4861.500 c	1628.000 a	1754.000 cd	557.000 d	133838.000 a	6784.000 ab
ARG_ME	1137.000 ab	10552.500 b	1977.000 bcd	4971.500 c	23813.000 a	2684.500 c	4936.500 bc	1131.000 d	1989.000 bc	6549.500 bc	3953.500 bcde	5480.500 bc	2315.000 b	8356.000 ab	1611.000 a	2292.500 bcd	2071.000 c	26090.500 f	4510.000 de
NZL_HA	993.000 b	2225.000 c	2425.500 abc	1393.500 d	2493.500 c	740.000 e	5509.500 bc	2586.500 b	2548.000 ab	11736.000 a	3247.000 de	7708.000 ab	2381.500 b	9945.000 a	2143.000 a	2587.500 bcd	4653.000 a	51892.500 cd	3190.500 e
NZL_MA	1025.500 b	5255.500 c	2718.500 ab	1184.000 d	5589.500 bc	596.000 e	3572.000 c	1338.500 cd	2992.000 ab	3704.000 c	2927.000 e	604.000 d	1332.000 b	4426.500 c	715.500 b	1030.500 d	1100.500 d	43773.000 cde	4842.000 cd
ITA_SP	1413.000 a	13931.000 ab	3030.500 a	12314.500 a	7895.500 b	6581.000 a	7727.000 b	2274.500 bcd	1012.000 c	4417.000 c	3562.000 cde	5051.000 bc	756.000 b	5013.500 c	1913.000 a	1580.000 cd	606.000 d	74524.500 b	6256.000 abc
Pr > F(Model)	0.016	0.002	0.017	<0.0001	<0.0001	<0.0001	0.007	0.013	0.025	0.028	0.016	0.005	0.004	0.001	0.006	0.027	<0.0001	<0.0001	0.003

	x.33	x.35	x.39	x.40	x.41	x.42	x.43	x.44	x.45	x.46	x.48	x.50	x.51	x.52	x.54	x.55	x.57	x.58	x.59	x.60
CLE_CV	9037.500 a	193505.000 a	21813.000 bc	2281.000 bc	7249.500 a	4956.500 bcd	2612.000 a	3232.000 bc	8011.500 b	6751.000 a	18924.000 a	3982.000 a	2672.500 bc	2120.000 a	2696.500 a	2229.000 a	14473.000 a	2771.500 bc	7770.500 bc	2620.000 bc
RSA_CO	2607.000 e	109065.500 b	16885.000 cd	3212.000 b	1731.000 cd	8697.500 abc	2373.500 ab	3306.000 bc	7034.000 b	8030.000 a	18138.500 a	1601.500 cd	2981.000 b	2054.000 a	930.000 d	2269.500 a	4954.500 d	4041.500 ab	10204.000 b	1246.000 d
RSA_WC	5240.500 bc	39132.500 d	26724.000 b	5406.000 a	5871.500 b	9680.000 ab	2085.000 ab	9580.500 a	9222.000 ab	5489.500 abc	15871.000 a	2640.000 b	4693.500 a	2080.500 a	1903.500 bc	2063.500 ab	10310.500 bc	3348.000 bc	9276.500 b	1866.500 cd
AUS_SE	5335.000 bc	101117.500 bc	42181.500 a	1731.500 cd	2430.000 c	12299.500 a	1883.500 bc	3611.000 b	11943.500 a	5356.000 abc	14455.000 a	2690.500 b	3250.500 b	2236.500 a	1868.000 c	1176.500 bcd	7656.500 cd	2955.000 bc	8443.500 b	1736.000 cd
ITA_VF	3784.000 cde	56356.500 cd	20453.000 bc	1857.000 cd	5990.000 b	2687.500 d	671.000 d	1963.500 bcde	2447.500 c	7343.500 a	16809.500 a	2723.500 b	2034.000 cd	2032.500 a	1625.500 c	2458.000 a	6884.500 cd	5362.500 a	14523.000 a	3868.000 a
ARG_ME	5110.500 bcd	174050.000 a	19348.000 c	870.500 de	2166.000 c	3650.500 cd	1375.500 c	1765.000 cde	6185.000 b	6644.000 a	16267.000 a	2390.500 bc	4054.500 a	2114.500 a	##########	1513.000 abc	8556.500 bcd	3288.000 bc	8021.000 bc	2785.500 b
NZL_HA	6591.000 b	168826.000 a	21712.500 bc	1422.500 cd	970.000 de	4167.000 bcd	1499.500 c	2503.000 bcd	8753.500 ab	3222.500 c	6288.500 b	2306.000 bc	1841.000 d	1668.000 a	2511.500 ab	397.500 d	12274.000 ab	2223.000 c	7816.500 bc	1148.000 d
NZL_MA	2621.500 e	75899.000 bcd	11576.000 d	264.000 e	1351.000 cde	1517.000 d	792.000 d	258.000 e	706.000 c	3627.500 bc	6332.000 b	692.000 d	704.500 e	510.000 b	463.500 d	504.500 cd	5537.500 d	2983.000 bc	7991.500 bc	2017.000 bcd
ITA_SP	3251.000 de	72897.500 bcd	16262.000 cd	924.500 de	442.000 e	3190.500 cd	2506.000 a	1257.000 de	809.000 c	6188.500 ab	13059.000 a	1802.500 bc	1268.500 de	1851.000 a	635.500 d	1929.500 ab	5387.000 d	2076.500 c	4444.500 c	1139.500 d
Pr > F(Model)	0.001	0.000	0.000	<0.0001	<0.0001	0.024	0.000	<0.0001	0.001	0.035	0.006	0.002	<0.0001	0.031	0.000	0.009	0.003	0.023	0.014	0.001

	x.61	x.63	x.64	x.68	x.69	x.70	x.71	x.73	x.74	x.75	x.76	x.77	x.78	x.84	x.85	x.86	x.87	x.88	x.90	x.94
CLE_CV	2983.000 ab	3553.500 bcd	30436.500 a	2191.000 b	9796.000 b	2469.000 a	10263.500 a	7206.500 a	2289.000 a	1811.500 bcd	4291.500 a	6176.500 a	10344.500 a	1086.500 c	7304.500 b	5370.000 a	1187.500 cd	4339.000 bc	67418.500 a	2541.000 bc
RSA_CO	2546.500 b	2242.000 ef	9885.000 de	1904.500 bc	5633.000 cd	2389.500 a	6918.000 bcd	2537.000 cd	893.500 cd	2243.000 abc	1499.500 cd	5822.500 ab	3158.500 de	575.500 de	3358.500 c	2151.000 cde	2917.500 a	3328.500 cd	45295.500 b	991.500 d
RSA_WC	2631.000 b	3890.500 bc	20964.000 bc	1780.500 bc	4977.500 de	2276.500 ab	6287.000 bcd	2574.000 cd	2026.500 ab	1882.000 bcd	937.500 d	3819.500 c	6125.500 bc	988.500 c	3313.500 c	4018.500 ab	2641.000 ab	1915.000 ef	50738.500 ab	2303.500 bc
AUS_SE	2757.500 b	3135.500 cde	14434.500 d	1309.000 d	4571.500 de	2042.000 abc	7589.000 bc	4091.500 bc	1737.500 ab	2620.000 ab	2517.000 bc	3771.000 c	4566.000 bcde	915.500 cd	2294.000 c	2682.500 bcd	2410.500 ab	2616.500 de	52242.000 ab	1482.500 cd
ITA_VF	1903.500 bc	1223.000 f	15820.500 cd	1539.000 cd	7409.500 c	1957.000 abc	7887.000 b	1634.000 d	1540.500 bc	3081.000 a	1187.500 d	4633.000 abc	3646.500 cde	330.000 e	12368.500 a	1449.000 de	1269.000 cd	4803.000 b	57712.500 ab	4375.500 a
ARG_ME	4239.500 a	5601.000 a	25046.000 ab	484.000 f	2025.500 fg	1655.000 bc	5591.500 cde	4857.500 b	1675.000 ab	1322.500 d	3058.000 ab	2852.000 c	5367.000 bcd	2702.000 a	1614.500 c	3416.000 bc	1720.500 bc	816.500 fg	60719.000 ab	855.500 d
NZL_HA	3309.500 ab	4453.500 ab	7933.500 ef	3984.500 a	353.500 g	274.500 d	716.500 f	3926.500 bc	866.500 cd	415.000 e	743.500 d	3051.000 c	7228.000 b	2716.000 a	7674.500 b	5309.000 a	752.000 cd	89.000 g	56236.000 ab	3117.500 b
NZL_MA	895.000 c	2591.500 de	6038.500 ef	1124.500 de	3468.000 ef	1816.500 abc	3541.000 e	3156.500 cd	882.000 cd	1571.000 cd	2603.000 bc	2887.500 c	2791.500 de	1780.500 b	4761.000 bc	1342.500 de	212.000 d	7321.000 a	22746.000 c	2149.500 bc
ITA_SP	1076.000 c	1042.000 f	2233.000 f	846.000 ef	16034.000 a	1364.000 c	4746.500 de	2285.000 d	763.000 d	2772.000 a	1588.000 cd	4044.000 bc	2142.000 e	277.000 e	2245.500 c	1260.000 e	217.000 d	3273.000 cd	45534.000 b	2838.500 b
Pr > F(Model)	0.009	0.000	<0.0001	<0.0001	<0.0001	0.001	0.000	0.001	0.004	0.002	0.002	0.021	0.002	<0.0001	0.001	0.000	0.002	<0.0001	0.018	0.001

	x.95	x.96	x.99	x.100	x.101	x.104	x.109	x.110	x.114	x.115	x.118	x.119	x.120	x.121	x.122	x.123	x.124	x.126	x.129	x.130
CLE_CV	2336.500 c	12343.000 ef	7178.500 bc	3899.500 a	7356.500 ab	10331.000 b	1968.000 a	6625.000 b	2198.500 a	3434.000 b	4371.000 cd	6501.500 a	3179.500 a	2801.500 a	6343.000 a	1433.500 b	7553.500 b	18652.500 a	2256.500 a	5399.000 a
RSA_CO	1423.500 cd	39226.500 a	3217.000 de	372.000 c	4632.500 de	10494.500 b	1065.000 cd	5709.500 bc	1067.000 bcd	2479.500 bc	13031.000 a	1154.500 ef	1424.000 bcd	636.500 c	4065.500 b	1445.000 b	17920.500 a	9685.000 c	1463.500 cd	3821.500 b
RSA_WC	807.000 d	21543.500 c	###########	1145.500 bc	5778.000 bcd	14048.000 ab	1244.000 bc	6024.000 bc	2612.500 a	2567.000 bc	7503.000 bc	3180.500 c	2414.000 ab	1282.500 bc	3763.500 b	1433.000 b	4849.500 bc	4902.000 de	1623.500 bc	3366.000 b
AUS_SE	2270.000 c	14801.000 de	11106.500 a	1406.500 bc	6480.000 abc	18535.000 a	831.500 cd	5160.000 bc	1685.500 abc	1236.000 cd	8704.500 b	1341.500 de	1864.500 bc	1143.500 bc	5653.000 a	2206.000 a	5395.500 bc	3833.500 def	2009.500 ab	3832.500 b
ITA_VF	7572.500 b	19095.500 cd	2220.000 e	1211.500 bc	8174.000 a	4153.500 c	1763.000 ab	10802.000 a	840.000 cd	3720.500 b	14129.000 a	2209.000 cd	890.000 cd	1104.000 bc	3228.500 b	423.500 c	5871.500 bc	6504.000 cd	971.000 e	5302.000 a
ARG_ME	9740.000 a	32341.000 b	6400.500 bcd	1029.000 bc	7754.000 a	10422.500 b	950.000 cd	7136.000 b	1937.500 ab	775.500 d	4808.500 bcd	4512.000 b	2920.000 a	1801.500 b	3868.500 b	1377.500 b	7438.000 b	2535.000 ef	1302.500 cde	3120.500 b
NZL_HA	1715.500 cd	1511.000 g	7968.500 ab	4988.500 a	4865.500 cde	13923.500 ab	1750.000 ab	3709.500 c	1802.000 abc	9065.500 a	4921.000 bcd	1075.000 ef	2318.500 ab	3149.500 a	742.000 c	2408.000 a	3095.500 cd	320.500 f	182.000 f	3810.500 b
NZL_MA	6996.500 b	15306.500 cde	4056.000 cde	987.000 bc	6015.000 bcd	2950.000 c	574.000 d	6623.500 b	533.000 d	1443.000 cd	8682.500 b	1610.000 de	1173.000 cd	681.000 c	3893.500 b	521.000 c	4433.500 bcd	5672.000 de	914.000 e	1316.500 c
ITA_SP	6842.000 b	7577.500 fg	4045.500 cde	2200.500 b	3300.000 e	2236.000 c	734.000 cd	3692.000 c	573.000 d	1605.000 cd	1448.500 d	313.500 f	723.000 d	403.500 c	3083.000 b	372.500 c	1100.000 d	14628.000 b	1122.500 de	2964.000 b
Pr > F(Model)	<0.0001	<0.0001	0.006	0.000	0.002	0.000	0.004	0.005	0.011	<0.0001	0.001	<0.0001	0.004	0.001	0.001	0.001	<0.0001	<0.0001	0.000	0.004

	x.131	x.133	x.139	x.143	x.145	x.146	x.148	x.149	x.154	x.155	x.157	x.158	x.159	x.160	x.161	x.164	x.166	x.168	x.169	x.171
CLE_CV	22465.500 b	1732.500 e	7430.000 a	8084.500 cd	5938.500 a	3378.000 a	1193.500 cd	2507.500 a	9993.500 a	5256.000 a	2660.000 cd	4423.500 bc	2305.000 a	43372.500 a	2682.500 a	1113.000 de	8245.500 a	13815.500 cd	5111.500 a	10149.500 a
RSA_CO	16904.000 c	7438.000 a	5018.000 bc	6069.000 e	3347.500 bc	1806.500 b	3615.000 a	1166.000 cd	8980.500 ab	1643.500 de	8865.000 ab	1521.000 d	1530.000 ab	22442.000 bc	2216.500 ab	5839.000 a	3875.500 c	18091.000 c	2229.500 bc	6578.000 ab
RSA_WC	3995.500 ef	5088.500 b	3885.000 cd	12257.000 b	7210.500 a	1607.500 b	2598.500 b	1395.000 bc	5032.500 bcd	2191.000 cd	6318.500 bc	6342.000 a	982.000 bc	18055.000 bcd	1658.000 abc	4213.500 b	3601.500 c	12132.500 de	2668.500 b	6200.500 ab
AUS_SE	6032.500 e	3501.500 cd	4859.500 bcd	11842.000 b	3194.500 bc	1780.500 b	2011.000 bc	1668.500 abc	7273.500 abc	3153.500 bc	5767.500 bc	5156.000 abc	1223.000 abc	22539.500 bc	1803.500 ab	2265.000 c	4056.000 bc	11870.500 de	2009.000 bc	8737.000 a
ITA_VF	10341.000 d	3794.000 c	5919.500 b	5944.000 e	2033.000 cd	749.500 c	1411.500 c	846.500 cd	7487.000 abc	782.500 ef	10929.500 a	1710.500 d	2164.000 a	26502.000 b	2368.000 ab	1525.500 cd	9805.500 a	54286.500 a	698.500 cd	7750.000 a
ARG_ME	10785.500 d	1681.000 e	5370.500 b	9533.000 c	4242.500 b	1971.000 b	1436.000 c	2243.000 ab	4866.500 bcd	3901.000 b	765.500 d	3601.500 c	757.000 bc	12963.000 bcd	2464.500 ab	844.000 de	9332.000 a	7950.500 e	1795.000 bcd	1992.500 bc
NZL_HA	2234.500 f	2373.500 de	3784.500 d	17023.500 a	2585.500 cd	1731.000 b	3662.500 a	1007.000 cd	428.000 d	3377.500 bc	2616.000 cd	5205.500 ab	123.000 c	3286.500 d	55.500 d	1471.500 d	3931.500 bc	17653.500 c	123.500 d	339.000 c
NZL_MA	40229.000 a	2125.500 e	4739.000 bcd	6517.000 de	2865.500 bcd	819.000 c	334.500 d	1397.000 bc	4020.000 cd	1691.000 de	2435.500 cd	1478.500 d	971.000 bc	11514.500 bcd	397.000 cd	1140.500 d	5564.500 b	13177.500 cd	3438.000 ab	1851.500 bc
ITA_SP	5243.500 ef	1476.000 e	2442.500 e	2813.500 f	1345.000 d	295.500 c	240.500 d	348.500 d	4243.500 bcd	255.000 f	625.500 d	475.500 d	1438.500 ab	10042.000 cd	1289.500 bcd	370.500 e	1697.000 d	28561.000 b	242.500 d	240.500 c
Pr > F(Model)	<0.0001	<0.0001	0.000	<0.0001	0.000	0.000	0.000	0.009	0.029	0.000	0.004	0.000	0.029	0.007	0.016	<0.0001	<0.0001	<0.0001	0.002	0.015

	x.172	x.173	x.175	x.178	x.179	x.180	x.182	x.183	x.184	x.185	x.186	x.187
CLE_CV	1629.500 bc	3794.000 ab	4535.500 b	3572.000 a	2201.000 def	7533.000 bc	2421.500 cd	2551.500 a	1318.500 bc	2949.000 cde	9750.500 cd	1787.000 bc
RSA_CO	5000.500 a	1669.000 cde	3375.000 bc	1336.500 cd	7618.000 a	12253.500 a	4654.000 b	1314.500 b	2503.500 a	8073.500 bc	4871.000 de	3611.500 ab
RSA_WC	2184.500 bc	3249.000 ab	2537.500 c	907.500 d	3960.500 cde	5330.000 cd	8742.000 a	596.500 bc	855.500 cd	13831.000 b	20298.500 ab	3872.500 a
AUS_SE	1700.500 bc	2746.000 bc	3392.000 bc	2225.000 bc	5353.500 abc	4705.500 cde	2923.500 c	634.500 bc	510.500 cd	21977.500 a	15144.500 bc	3107.000 ab
ITA_VF	2169.000 bc	1193.500 de	3017.500 bc	558.500 d	4193.500 cd	11008.500 ab	1464.500 de	1395.000 b	1984.500 ab	313.000 de	3121.000 de	1079.500 c
ARG_ME	1455.500 cd	4412.500 a	4440.500 b	3202.500 ab	4613.000 bcd	1835.500 de	1472.500 de	888.000 bc	71.500 d	7060.500 bcd	10125.500 cd	2054.500 abc
NZL_HA	2572.000 b	2428.500 bcd	6359.500 a	2133.000 bc	6926.000 ab	7380.500 bc	1899.500 cde	2287.500 a	2263.000 a	1882.000 cde	24333.000 a	649.000 c
NZL_MA	395.000 e	1304.500 de	563.500 d	599.500 d	1650.000 ef	9058.000 abc	5804.000 b	245.500 c	2537.000 a	83.000 e	5719.500 de	304.000 c
ITA_SP	513.500 de	418.000 e	451.500 d	295.000 d	342.000 f	673.500 e	943.000 e	1103.500 bc	63.000 d	1792.000 cde	1330.500 e	1146.500 c
Pr > F(Model)	<0.0001	0.001	0.000	0.001	0.001	0.003	<0.0001	0.003	0.000	0.001	0.001	0.016

Table S9. LS mean and group (indicated by the letter) obtained by the ANOVA one-way and Tukey HSD post hoc test for the significant phenolic compounds for the Sauvignon Blanc wines (α = 0.05).

	x1	x2	x3	x4	x8	x9	x11	x12	x14	x15	x16	x17	x18	x19	x20	x22	x23	x24	x26	x27
AUS_SE	12316.500 a	9440.000 a	7330.500 a	5487.000 ab	8753.500 b	4145.000 b	5832.000 a	4414.000 a	3239.000 a	3870.000 ab	8427.000 bc	3667.000 abc	3799.500 ab	5160.500 a	26140.000 cd	3800.500 ab	7221.000 ab	67614.500 bcd	8084.000 abc	5996.000 ab
CLE_CV	15772.500 a	3493.000 a	2831.500 b	2778.500 b	11129.000 b	1917.000 b	4017.000 ab	3425.500 a	1575.000 bc	3517.500 abc	35034.000 a	4195.000 ab	5513.000 ab	2774.000 ab	18054.500 cde	5248.000 a	6233.500 bc	123713.000 a	11656.500 a	6954.000 ab
RSA_WC	9430.500 a	5879.500 a	5237.500 ab	4385.500 b	9116.000 b	2575.500 b	4804.000 a	4860.500 a	1801.000 bc	3248.500 abcd	8202.000 bc	3208.500 abc	6386.500 a	3823.000 ab	19727.000 cde	1887.500 bcd	6702.000 bc	51450.000 cd	7111.000 abc	4566.000 ab
ITA_SP	11302.500 a	6556.000 a	5599.000 ab	11967.000 a	21730.000 a	12387.000 a	5625.000 a	3793.500 a	1299.500 bc	4376.500 a	11231.500 bc	5754.000 a	5097.500 ab	3553.500 ab	52441.000 a	1849.000 bcd	10336.500 a	40362.000 cd	5226.000 bc	2723.000 b
ARG_ME	10507.500 a	10460.500 a	4506.000 ab	6520.500 ab	22657.000 a	4368.000 b	4247.500 ab	3615.000 a	2225.500 ab	4575.500 a	11047.000 bc	3757.500 abc	4219.000 ab	1130.500 b	17901.000 cde	3234.000 abc	10506.500 a	83491.000 abc	9928.500 ab	6728.500 ab
ITA_VF	9446.000 a	5310.500 a	4895.500 ab	2422.500 b	5248.500 b	1994.500 b	5593.000 a	3384.500 a	873.500 c	1857.500 cd	17303.000 b	2662.500 bc	3597.500 b	1223.500 b	42527.500 b	1468.500 cd	6314.500 bc	26393.000 d	3539.500 c	2887.500 b
RSA_CO	10377.000 a	5911.500 a	5253.500 ab	2511.000 b	9591.000 b	1432.500 b	4307.000 ab	3619.000 a	1828.000 bc	2182.500 bcd	7112.000 c	2344.500 bc	5677.000 ab	3405.500 ab	27499.000 c	1018.000 d	5667.500 bc	60610.500 cd	6948.000 abc	4914.000 ab
NZL_MA	14055.000 a	10188.500 a	7156.000 a	1895.500 b	10073.000 b	1085.500 b	2440.500 b	833.500 b	2139.000 abc	1385.500 d	6893.000 c	1479.500 c	4918.500 ab	2268.000 ab	13260.500 e	1011.500 d	4259.000 bc	64761.500 bcd	7265.000 abc	6431.000 ab
NZL_MR	16005.500 a	7633.000 a	4306.000 ab	1765.500 b	7995.000 b	932.500 b	2507.500 b	358.000 b	2297.500 ab	1490.500 d	6499.500 c	1616.500 bc	5107.500 ab	2520.000 ab	16585.500 de	1936.000 bcd	3345.000 c	112697.000 ab	10949.500 ab	9071.500 a
Pr > F(Model)	0.022	0.048	0.017	0.006	0.000	0.001	0.001	0.001	0.002	0.000	<0.0001	0.003	0.042	0.033	<0.0001	0.000	0.000	0.000	0.011	0.013

	x28	x29	x30	x31	x32	x35	x36	x37	x39	x40	x41	x42	x43	x44	x45	x49	x50	x51	x54	x56
AUS_SE	43383.000 a	2541.000 b	8200.000 a	2867.000 c	6870.500 a	23408.500 abcd	1576.500 ab	6850.500 a	3592.500 a	2006.500 bc	6390.000 b	25546.000 a	4976.000 c	7814.000 ab	2132.500 bc	18049.500 ab	13066.000 ab	1521.500 ab	3179.500 bc	30876.000 ab
CLE_CV	14515.500 bcd	1570.000 b	2984.500 cd	2708.500 c	4785.000 a	12498.500 bcd	1829.000 ab	5355.500 a	2302.500 bc	4804.500 a	8315.500 b	12850.000 b	7138.000 bc	8493.500 a	3894.500 ab	13712.500 abc	17096.500 a	2113.500 ab	1989.500 bc	31188.000 ab
RSA_WC	16839.500 bcd	3383.000 b	5374.500 b	1545.000 c	7251.500 a	21314.000 abcd	2297.500 a	6527.500 a	1420.500 de	5204.500 a	8165.500 b	13282.500 b	4520.500 c	6185.000 abc	4042.500 a	19567.000 a	10152.500 ab	2363.000 a	2196.500 bc	30035.500 ab
ITA_SP	17867.500 bc	2157.500 b	8328.500 a	18422.500 a	3985.000 a	9649.000 d	1740.000 ab	4677.500 a	1679.000 bcde	3935.500 ab	5452.000 b	12886.500 b	21851.000 a	7180.000 abc	4579.500 a	15777.500 abc	10885.500 ab	1651.000 ab	1169.500 c	27332.500 ab
ARG_ME	14485.500 bcd	8377.000 a	1142.500 d	2240.000 c	9087.500 a	31166.500 a	2012.500 a	6801.000 a	871.000 e	3116.000 ab	25388.500 a	12201.000 b	7304.000 bc	6673.500 abc	2074.000 bc	16117.000 abc	9760.500 ab	1479.500 ab	10719.500 a	36013.000 a
ITA_VF	21878.500 b	2482.000 b	1592.500 d	2667.000 c	4436.000 a	11237.500 cd	1349.500 ab	4254.500 a	2230.500 bcd	2365.500 bc	5460.000 b	9131.500 b	10831.500 b	8284.000 a	2091.500 bc	13219.000 abc	8166.500 ab	1488.500 ab	8007.500 ab	25462.000 ab
RSA_CO	13815.500 bcd	2310.000 b	4240.500 bc	1559.000 c	6976.500 a	24312.500 abc	1705.000 ab	5219.500 a	1564.000 cde	2895.000 abc	4002.500 b	7053.000 b	3120.500 c	4459.500 bcd	2797.000 abc	14562.500 abc	8568.000 ab	1127.500 ab	1951.500 bc	17552.500 b
NZL_MA	10641.000 cd	2603.500 b	2181.500 d	2549.000 c	10236.500 a	31273.500 a	811.500 b	4089.000 a	2442.500 b	493.500 c	5636.000 b	4040.500 b	3471.500 c	2398.000 d	629.000 c	8026.500 c	8264.000 ab	469.000 b	1417.000 bc	14557.500 b
NZL_MR	8143.500 d	2135.000 b	2319.000 cd	5806.500 b	8880.000 a	26285.000 ab	790.500 b	4892.000 a	1976.500 bcd	275.500 c	5119.000 b	4426.500 b	3692.000 c	3554.000 cd	349.000 c	8986.000 bc	7563.500 b	431.500 b	389.500 c	16168.000 b
Pr > F(Model)	<0.0001	<0.0001	<0.0001	<0.0001	0.039	0.001	0.009	0.033	<0.0001	0.000	<0.0001	0.001	<0.0001	0.001	<0.0001	0.010	0.046	0.016	0.002	0.009

	x58	x59	x62	x66	x67	x68	x71	x73	x79	x82	x83	x84	x86	x87	x89	x90	x91	x92	x94	x96
AUS_SE	2428.000 a	16719.000 bcd	8355.500 a	2895.500 abcd	2695.500 a	3339.500 a	4427.000 ab	5696.000 a	1574.000 abc	9128.500 ab	5963.500 a	1393.500 b	8277.000 bc	5222.500 a	2877.000 ab	8716.500 bc	2629.000 cd	1218.500 bcd	6948.000 ab	4916.000 a
CLE_CV	1184.000 ab	9425.000 cde	7819.500 a	5031.500 a	1519.500 a	1948.000 b	4778.500 ab	1760.500 bcd	2457.500 a	4352.500 bc	2171.500 b	1934.000 b	5098.000 cd	3680.000 ab	3615.000 a	9906.000 abc	5713.500 a	1222.000 bcd	5075.000 ab	943.000 d
RSA_WC	1601.500 ab	11287.500 cde	9437.000 a	4656.500 ab	2753.500 a	3141.500 a	3710.500 b	2284.500 bc	2328.500 ab	3182.000 c	2052.500 b	2493.000 b	3269.000 cd	2317.000 bc	2730.000 ab	6032.000 c	1303.500 e	1821.000 bc	4525.000 ab	2100.500 bc
ITA_SP	2278.500 a	18184.500 bc	6283.000 a	3238.000 abcd	2590.000 a	1610.000 bc	3847.500 b	2774.500 b	2090.000 ab	5219.500 bc	566.500 b	900.500 b	4964.000 cd	3610.500 ab	3348.500 a	1137.500 c	3466.000 bc	1755.500 bc	7533.000 a	4399.500 a
ARG_ME	1070.000 ab	6913.000 de	8265.500 a	2348.000 bcde	2314.000 a	1599.000 bc	7043.500 a	705.500 d	1978.000 ab	4550.500 bc	861.000 b	6553.500 a	4059.500 cd	2694.000 bc	2833.000 ab	4034.500 c	1298.500 e	3271.500 a	3319.000 ab	1476.000 cd
ITA_VF	239.500 b	1859.000 e	4738.500 a	4180.500 abc	3028.500 a	871.500 c	3983.000 b	1736.500 bcd	1085.500 bcd	5359.500 bc	4817.000 a	474.500 b	1703.000 d	2894.000 bc	3283.000 a	17391.500 ab	3951.500 b	2161.500 ab	3645.500 ab	1884.000 bcd
RSA_CO	1900.500 ab	19097.500 bc	6096.000 a	1832.000 cde	1484.000 a	2468.500 ab	3622.000 b	1172.000 cd	1449.000 abc	4131.000 bc	4821.500 a	1318.500 b	13086.000 ab	1749.000 c	2233.000 abc	6873.000 c	1883.500 de	1577.000 bcd	2994.500 ab	3824.000 a
NZL_MA	2567.000 a	31207.000 a	4543.000 a	887.500 de	1421.000 a	769.000 c	4220.000 b	1977.000 bcd	287.500 cd	14007.000 a	1374.000 b	1187.000 b	16361.500 a	2235.500 bc	1167.000 c	6851.500 c	1509.500 de	961.000 cd	2806.000 b	2666.500 b
NZL_MR	1918.500 ab	25390.000 ab	4575.500 a	332.500 e	1358.000 a	836.000 c	5446.000 ab	1651.500 bcd	148.000 d	11989.000 a	4319.500 a	1570.500 b	14625.000 ab	2771.000 bc	1446.000 bc	19357.500 a	3672.000 bc	525.500 d	3782.000 ab	4365.500 a
Pr > F(Model)	0.022	<0.0001	0.036	0.000	0.035	<0.0001	0.016	<0.0001	0.000	0.000	<0.0001	<0.0001	<0.0001	0.001	0.001	0.001	<0.0001	0.000	0.025	<0.0001

	x97	x99	x101	x103	x106	x108	x110	x112	x113	x115	x116	x117	x118	x122	x123	x124	x125	x126	x127	x128
AUS_SE	3459.000 c	26311.500 a	2291.500 bc	3046.500 bc	3214.500 a	11279.000 ab	4935.000 ab	6871.500 ab	7539.500 a	5163.000 bc	18357.500 ab	2797.500 a	1958.500 ab	10032.000 ab	6051.000 ab	1332.000 ab	4893.000 a	2024.500 ab	4250.000 ab	1370.000 bcd
CLE_CV	5069.500 a	9445.000 cd	3528.500 b	3707.000 b	3267.000 a	8341.000 b	5826.000 ab	5414.500 abc	1353.000 b	7746.500 a	12758.000 abc	662.500 c	2032.500 ab	7994.000 ab	5269.000 ab	1693.000 a	3844.500 ab	1947.500 ab	1360.500 c	2746.000 a
RSA_WC	1846.000 e	21803.500 b	2130.000 bc	3871.000 b	1732.000 b	8918.500 b	6485.500 a	2530.000 c	7126.500 a	4479.000 bc	8594.000 abc	2012.500 ab	2626.000 ab	13391.500 ab	8400.000 ab	2153.500 a	1430.500 cd	1555.000 ab	2026.000 bc	1035.000 cde
ITA_SP	3351.000 cd	8089.000 d	10000.000 a	5696.500 a	1492.000 b	19578.500 a	6096.500 ab	7234.000 a	1822.000 b	6260.000 ab	20623.000 a	1028.500 c	3234.000 a	3109.000 b	1700.500 b	1128.000 ab	3450.000 abc	3058.500 a	1241.500 c	975.000 de
ARG_ME	1325.000 e	9970.500 cd	1921.000 c	2206.000 c	1108.500 bc	10634.500 ab	4519.000 ab	1991.500 c	942.000 b	6350.500 ab	4643.000 c	1065.000 c	1581.000 ab	15490.500 a	10150.000 a	1140.500 ab	468.000 d	1106.500 b	1463.000 c	261.500 e
ITA_VF	3834.000 bc	7127.000 d	1750.000 c	2149.000 c	790.000 bc	12721.500 ab	4018.500 b	3663.500 abc	767.500 b	4328.500 bc	12119.500 abc	1036.500 c	1973.000 ab	9464.000 ab	5794.500 ab	1202.500 ab	2369.000 bcd	1370.000 b	4888.000 a	518.000 de
RSA_CO	1607.500 e	10809.000 cd	1655.000 c	2910.000 bc	1216.500 bc	6788.500 b	4188.500 ab	1671.000 c	1310.500 b	3393.500 c	3543.000 c	2503.000 a	1411.000 ab	9379.500 ab	5370.000 ab	1010.500 ab	261.500 d	935.000 b	4652.500 a	2067.000 ab
NZL_MA	2193.500 de	12671.000 c	1391.500 c	278.000 d	225.500 c	7250.500 b	1029.500 c	1903.500 c	2021.000 b	3070.000 c	5883.500 bc	1099.000 c	924.000 b	17344.000 a	11219.000 a	260.000 b	512.500 d	423.000 b	1221.000 c	1957.500 abc
NZL_MR	4733.500 ab	9016.500 cd	1457.500 c	94.000 d	1011.500 bc	6890.000 b	685.000 c	3021.500 bc	565.500 b	3527.500 c	10063.000 abc	1256.500 bc	923.000 b	7401.000 ab	4064.500 ab	175.000 b	1163.500 cd	915.500 b	2227.000 bc	1232.500 bcd
Pr > F(Model)	<0.0001	<0.0001	<0.0001	<0.0001	<0.0001	0.014	<0.0001	0.001	<0.0001	0.000	0.007	<0.0001	0.035	0.009	0.027	0.004	0.000	0.004	0.000	<0.0001

	x129	x132	x137	x138	x139	x142	x143
AUS_SE	3280.000 a	2681.000 ab	6812.000 ab	9091.500 bc	47055.500 a	5376.500 ab	16810.500 b
CLE_CV	1884.500 abc	3996.000 a	3919.500 bc	5515.500 cd	10919.500 bc	2608.000 bc	10704.000 c
RSA_WC	1541.500 bc	3959.500 a	8027.000 a	897.500 ef	43542.500 a	8029.000 a	1504.500 d
ITA_SP	3031.000 ab	3398.500 ab	2824.500 bc	1520.000 ef	8717.000 bc	1964.000 bc	1452.000 d
ARG_ME	1103.500 c	3511.500 ab	675.000 c	6115.000 cd	7852.000 bc	345.500 c	18075.500 ab
ITA_VF	1667.500 abc	2081.000 ab	1923.500 c	10187.500 ab	7250.500 bc	781.000 c	7577.000 c
RSA_CO	652.000 c	2059.500 ab	2642.000 c	619.500 f	13963.000 b	1815.000 bc	1139.000 d
NZL_MA	1010.500 c	1474.000 ab	1278.000 c	13436.500 a	11697.500 bc	996.000 c	22592.500 a
NZL_MR	1614.500 abc	1116.000 b	6870.000 ab	4371.000 de	5711.000 c	5012.500 ab	5853.000 cd
Pr > F(Model)	0.002	0.012	0.000	<0.0001	<0.0001	0.000	<0.0001

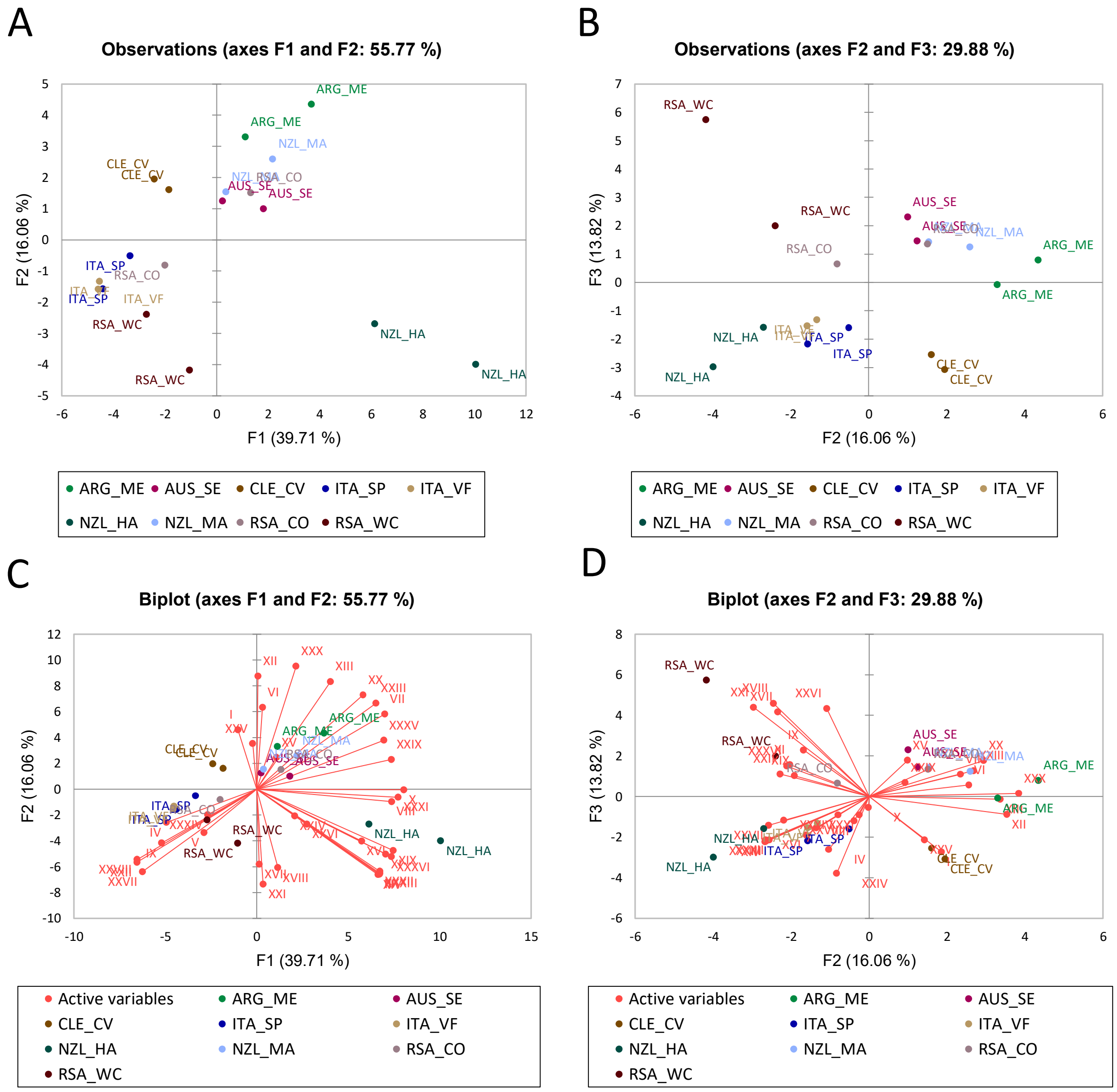

Figure S2. Principal component analysis on volatile compounds for Chardonnay data. Graphs A (observation) and C (biplot) show PC1 vs PC2. Graphs B (observation) and C (biplot) show PC2 vs PC3.

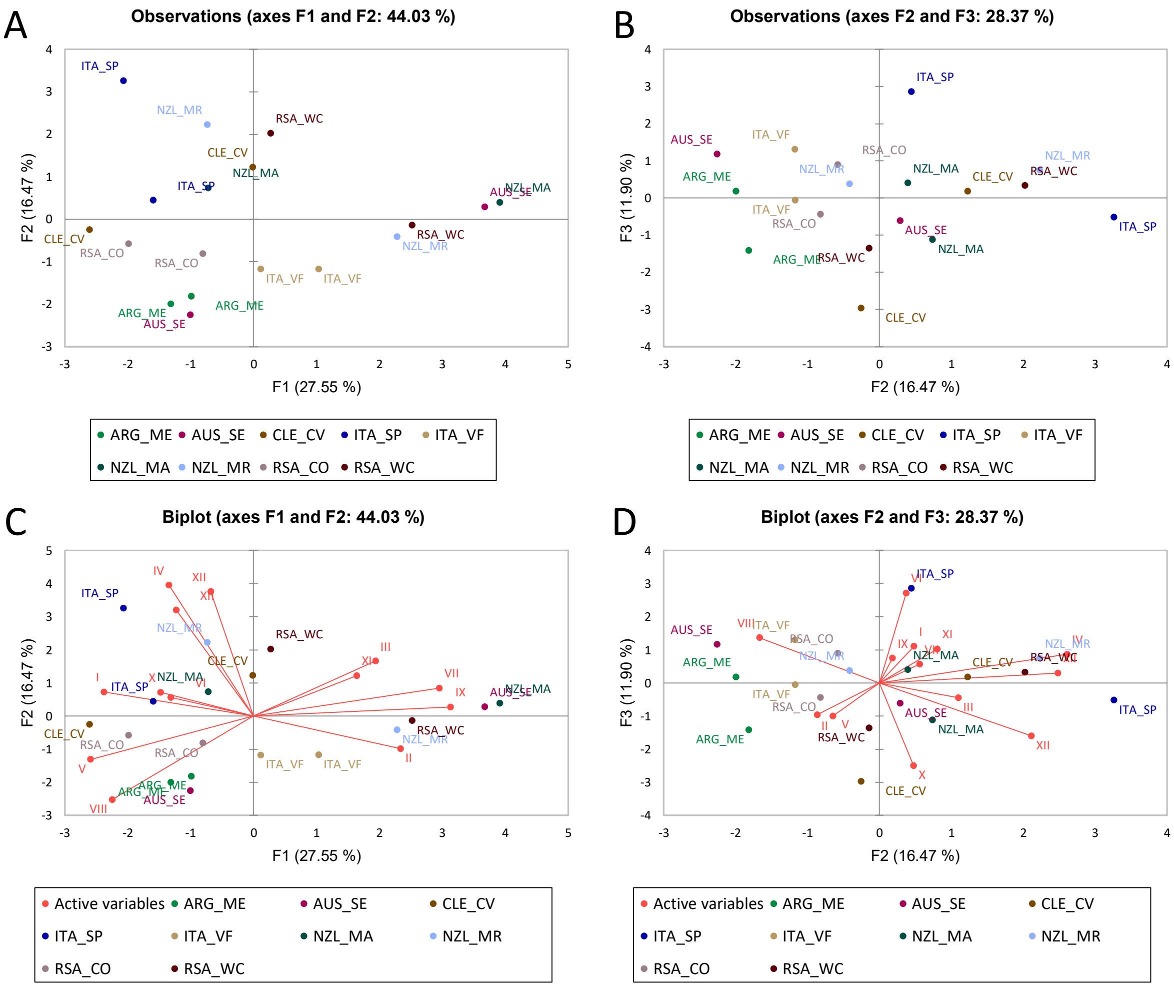

Figure S3. Principal component analysis on volatile compounds for Sauvignon Blanc data. Graphs A (observation) and C (biplot) show PC1 vs PC2. Graphs B (observation) and D (biplot) show PC2 vs PC3.

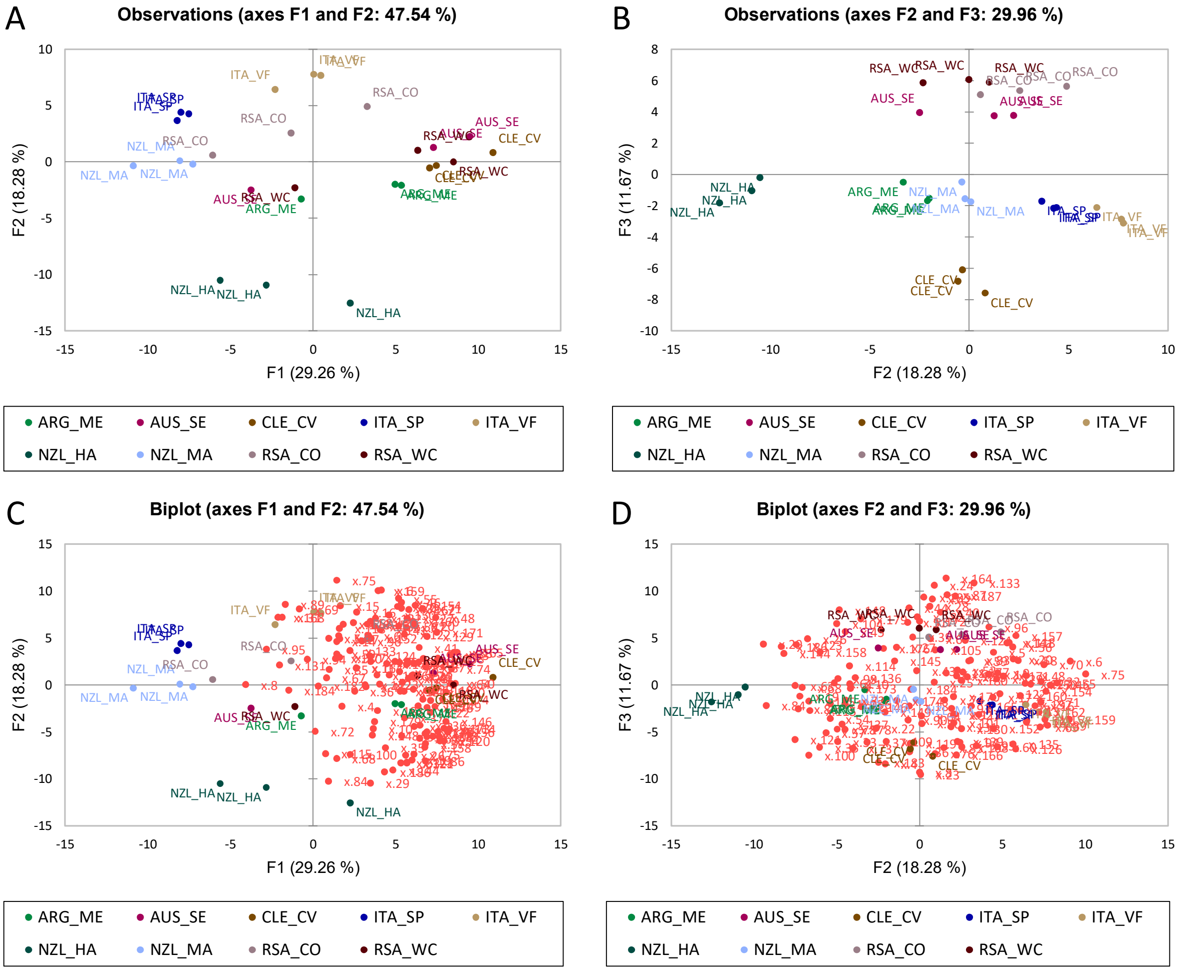

Figure S4. Principal component analysis on phenolic compounds for Chardonnay data. Graphs A (observation) and C (biplot) show PC1 vs PC2. Graphs B (observation) and D (biplot) show PC2 vs PC3.

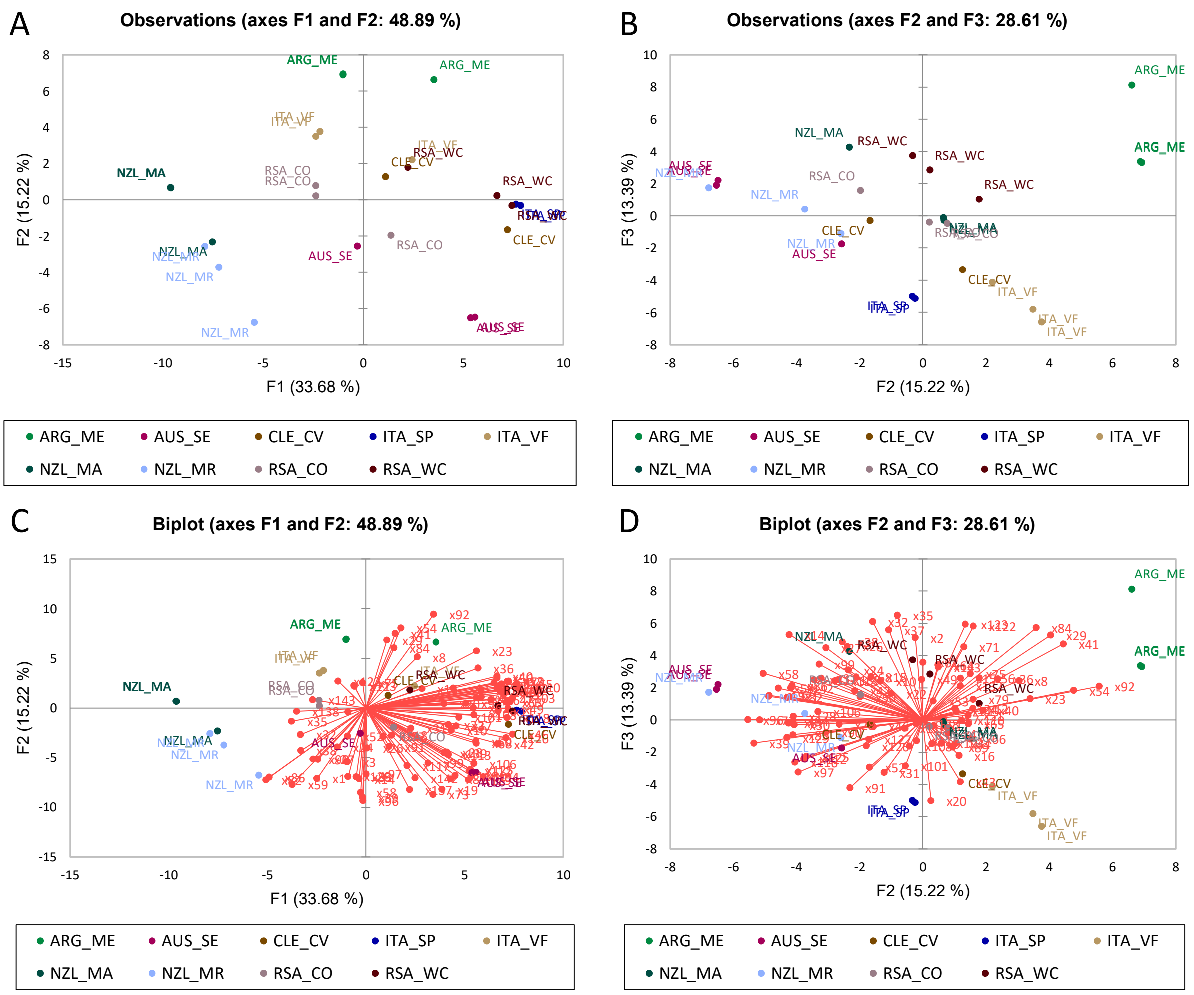

Figure S5. Principal component analysis on phenolic compounds for Sauvignon data. Graphs A (observation) and C (biplot) show PC1 *vs* PC2. Graphs B (observation) and D (biplot) show PC2 *vs* PC3.

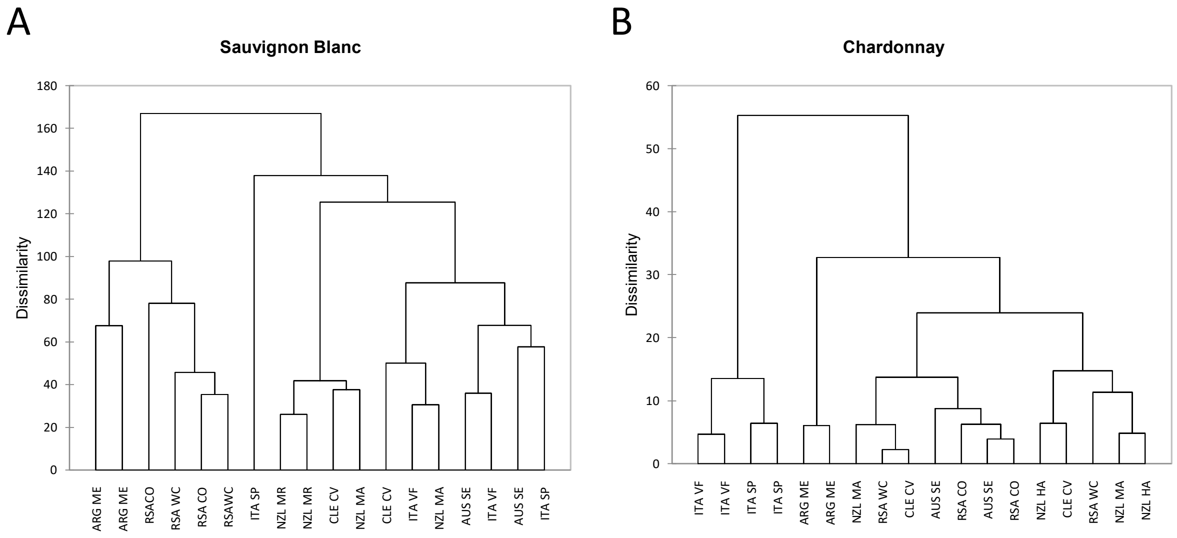

Figure S6. Agglomerative hierarchical clustering on sensory analysis data for (A) Sauvignon Blanc wines and (B) Chardonnay wines.

Table S10. Tentative compound assignment for Chardonnay wines.

Code	Name	Mean RT1 (min)	Mean RT2 (sec)	RI lib	Base mass
I	Ethyl Acetate	4.42	1.38	888	43
II	NI	4.92	2.07		
III	NI	5.59	1.93		
IV	4-Amino-1-butanol	5.76	1.58	1687	45
V	NI	14.97	1.76		
VI	3-Pentanol, 2-methyl- (IS)	15.52	1.34	1121	59
VII	NI	17.14	1.78		
VIII	Ethylamine	21.77	0.85	715	44
IX	2-Pentanol, 3-methyl-	23.11	1.38	1181	45
X	Butyl lactate	23.11	1.2	1514	45
XI	1-Propanol, 3-amino-	23.15	1.66	1555	44
XII	2,3-Butanediol	23.15	1.33	1543	45
XIII	Propanoic acid, 2-hydroxy-, ethyl ester (or methyl ester)	23.15	0.97	1347	45
XIV	Octanoic acid, ethyl ester	26.24	0.76	1435	88
XV	Acetic acid	27.61	1.28	1449	
XVI	NI	27.61	1.8		
XVII	2,4-Hexadienoic acid, ethyl ester	29.57	0.09	1501	95
XVIII	NI	29.61	0.43		
XIX	NI	29.70	0.73		
XX	NI	34.74	0.89		88
XXI	NI	34.74	1.13		88
XXII	Decanoic acid, ethyl ester	34.91	0.53	1638	88
XXIII	Butanedioic acid, diethyl ester	36.41	2.19	1680	101
XXIV	NI	37.16	1.45		93
XXV	Naphthalene, 1,2-dihydro-1,1,6-trimethyl-	38.21	1.9	1732	157
XXVI	Acetic acid, 2-phenylethyl ester	41.21	1.72	1813	104
XXVII	Hexanoic acid, 2-phenylethyl ester	41.25	2.18	2162	
XXVIII	NI	44.42	1.72		
XXIX	Benzene-butanal	44.84	2.1	1792	
XXX	Phenylethyl Alcohol	44.84	0.85	1906	91
XXXI	Sorbic Acid	52.39	2.14	2150	
XXXII	NI	52.76	1.52		
XXXIII	Naphthalene, decahydro-4a-methyl-1-methylene-7-(1-methylethylidene)	52.89	1.51	1682	
XXXIV	n-Decanoic acid	55.35	0.91	2276	60
XXXV	2,4-Di-tert-butylphenol	55.85	1.42	2318	191

Table S11. Tentative compound assignment for Sauvignon Blanc wines.

Code		RT I dim	RT II dim	RI	Base mass
I	1-octen-3-ol	28.23	1.21	1450	57
II	2-sec-butyl-3-methoxypyrazine	30.00	1.78	1427	137
III	3-mercapto-hexanol	43.26	0.98	1853	55
IV	3-mercapto-hexyl acetate	38.64	1.39	1735	43
V	4-mercapto-4-methyl-2-pentanone	24.85	1.25	1383	43
VI	acetic acid, hexyl ester	20.41	1.97	1272	43
VII	benzaldehyde	30.85	1.11	1520	51
VIII	butanoic acid, ethyl ester	10.41	1.70	1095	41
IX	Ethyl cinnamate	52.47	1.19	2012	131
X	furfuryl mercaptan	27.10	1.11	1431	81
XI	trans-2-nonenal	31.44	1.56	1542	41
XII	α-Terpineol	37.99	1.32	1697	59
XIII	β-Damascenone	42.18	1.58	1823	69

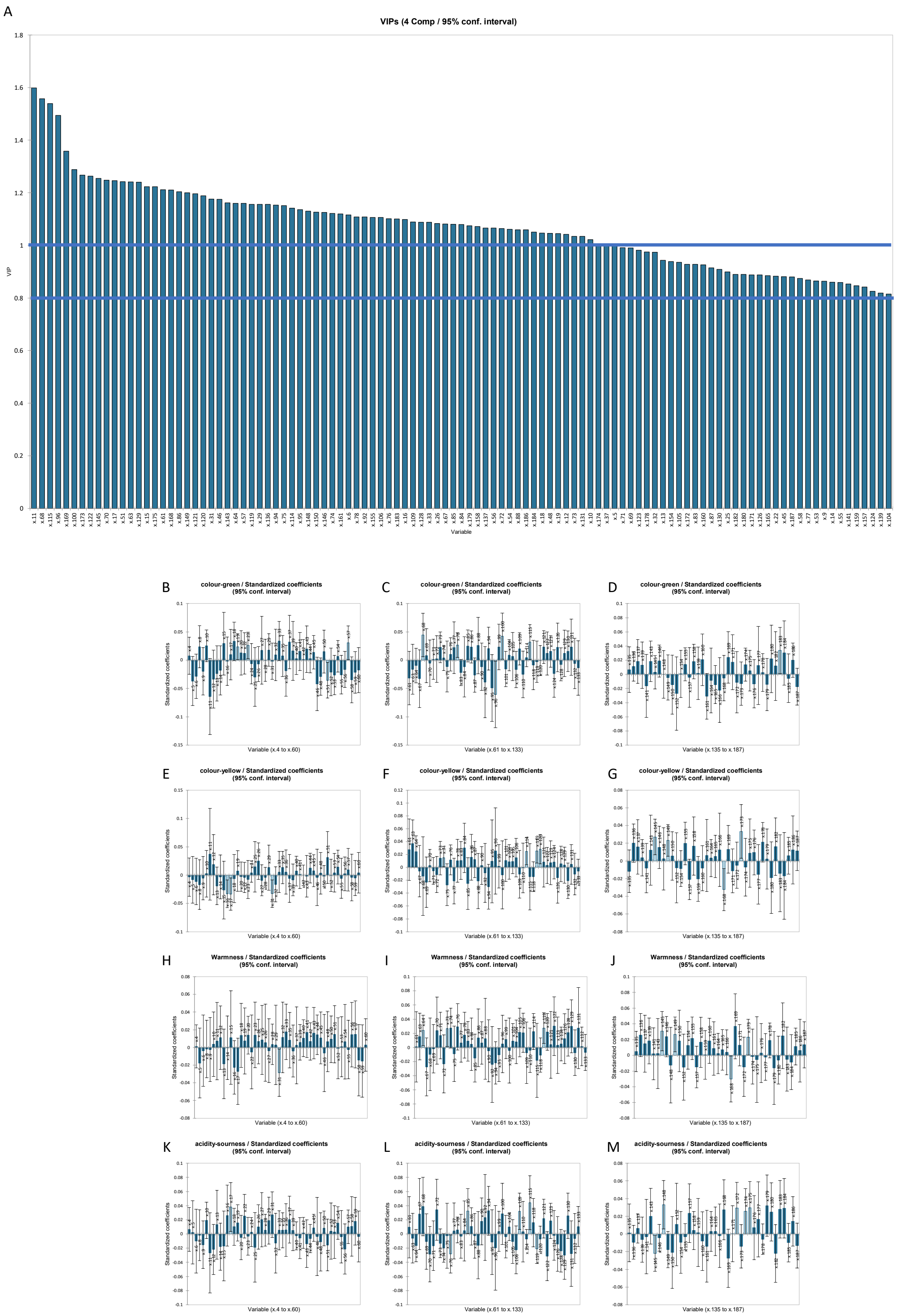

Figure S7. PLS-R for the non-volatile phenolic compound with the visual and gustatory data.

The figure A shows the different VIP while from figure B to M show the standardized coefficient effect for the variables in the regression.

Table S12. PAC compounds observed in the LC-MS analysis.

m/z	RT	Number	Compounds
579.1	29.6	x.1	Dimers (PC)
579.1	30.9	x.2	Dimers (PC)
579.1	34.5	x.3	Dimers (PC)
579.1	37.8	x.4	Dimers (PC)
595.1	27.6	x.5	Dimers OH (PD)
595.1	30.9	x.6	Dimers OH (PD)
611.1	17.2	x.7	Dimers 2-OH (PD)
867.2	35.6	x.8	Trimers (PC)
883.2	18.0	x.9	Trimers OH (PD)
1153.3	22.8	x.10	c-tetramers (PC)
1155.3	28.1	x.11	c-tetramers (PC)
1155.3	36.7	x.12	c-tetramers (PC)
1155.3	37.0	x.13	tetramers (PC)
1169.3	22.2	x.14	c-OH-tetramers (PD)
1171.3	30.9	x.15	OH-tetramers (PD)
1201.3	22.1	x.16	c-3-OH-tetramers (PD)
1441.4	24.6	x.17	c-pentamers (PC)
1443.4	34.5	x.18	pentamers (PC)
1443.4	42.0	x.19	pentamers (PC)
1457.4	34.6	x.20	c-OH-pentamers (PD)

Table S13. ANOVA significant variables for the PAC dataset on Chardonnay wines.

	x.1	x.4	x.5	x.6	x.8	x.9	x.10	x.12	x.13	x.14	x.16	x.17	x.18	%c-5
RSA_WC	178415.317 b	0.000 a	7284.227 bcd	39354.020 b	2211.333 b	4703.223 b	3910.700 bc	225.000 b	352.913 b	3390.973 ab	3752.113 bc	1434.070 d	118.333 ab	90.692 b
ARG_ME	27259.367 a	24547.507 b	8490.010 d	32132.043 ab	0.000 a	691.290 a	5460.787 c	0.000 a	50.867 ab	5342.070 b	5016.947 c	1269.920 cd	269.030 b	81.811 b
AUS_SE	49491.913 a	3300.333 a	2782.370 abc	12401.480 ab	730.497 ab	757.237 a	2916.613 abc	0.000 a	163.787 ab	3155.677 ab	3232.573 abc	1012.770 bcd	99.757 ab	88.357 b
RSA_CO	122666.467 ab	2319.147 a	8074.317 cd	17162.030 ab	1460.363 ab	806.797 a	2835.747 abc	0.000 a	127.523 ab	3132.437 ab	2967.887 abc	377.000 ab	0.000 a	100.000 b
NZL_MA	60318.637 a	7568.437 ab	2795.063 abc	13330.470 ab	315.803 a	371.590 a	1714.973 ab	0.000 a	50.030 ab	2285.800 a	2130.507 ab	746.313 abcd	201.957 b	79.146 b
CLE_CV	22117.520 a	2346.413 a	2361.745 ab	23498.783 ab	0.000 a	360.603 a	940.203 ab	0.000 a	0.000 a	3870.087 ab	3890.137 bc	345.493 ab	114.877 ab	74.937 ab
NZL_HA	14039.527 a	1811.803 a	0.000 a	10525.227 a	0.000 a	1588.427 a	2063.347 ab	0.000 a	54.087 ab	1767.240 a	1221.077 a	692.450 abc	120.470 ab	85.120 b
ITA_SP	10074.763 a	743.330 a	1946.107 ab	15688.360 ab	308.453 a	1379.810 a	288.620 a	0.000 a	0.000 a	2698.583 a	2596.027 ab	77.947 a	0.000 a	89.446 b
ITA_VF	36268.657 a	655.333 a	1764.203 ab	7048.747 a	0.000 a	340.503 a	278.240 a	0.000 a	0.000 a	2782.380 a	2051.090 ab	143.943 a	117.617 ab	48.656 a
Pr > F(Model)	0.001	0.016	0.000	0.013	0.001	0.000	0.000	0.017	0.013	0.006	0.000	<0.0001	0.004	0.000

Table S14. ANOVA significant variables for the PAC datasets on Sauvignon Blanc.

	x.1	x.2	x.6	x.9	x.10	x.12	x.14	x.16	x.17	%c-4	%c-4-OH	%c-5
AUS_SE	127045.440 a	3768.343 ab	30170.375 ab	8004.463 b	3185.723 b	51.590 ab	4927.650 abc	4891.107 ab	1161.653 b	90.449 b	80.776 a	84.801 ab
NZL_MR	141814.823 a	2566.490 a	22856.750 ab	1575.873 a	1485.787 ab	0.000 a	6012.607 c	5771.007 b	701.210 ab	92.929 b	88.412 a	79.156 ab
RSA_CO	88949.160 a	2602.310 a	26601.807 ab	2078.247 a	2074.577 ab	0.000 a	4368.313 abc	3510.023 ab	822.667 ab	100.000 b	84.281 a	83.621 ab
ITA_SP	170645.760 a	2506.980 a	21152.315 ab	2853.865 a	6371.975 c	266.470 b	2350.385 ab	2405.355 ab	2216.320 c	86.316 b	76.737 a	92.012 b
ARG_ME	9404.573 a	3328.723 ab	37299.797 ab	478.837 a	204.515 a	0.000 a	4637.760 abc	3075.270 ab	308.257 ab	100.000 b	79.726 a	52.687 a
RSA_WC	24967.883 a	6104.967 b	36295.617 ab	3879.600 a	0.000 a	63.433 ab	3895.837 abc	3052.743 ab	559.840 ab	0.000 a	81.665 a	89.361 b
NZL_MA	34768.583 a	2333.213 a	12400.140 a	0.000 a	754.437 ab	0.000 a	5347.813 bc	4164.557 ab	340.247 ab	100.000 b	87.913 a	73.926 ab
CLE_CV	7384.265 a	2059.000 a	46745.180 b	858.790 a	0.000 a	242.230 ab	2410.560 ab	2023.150 ab	334.120 ab	0.000 a	75.673 a	69.926 ab
ITA_VF	33380.217 a	1173.200 a	8385.830 a	621.337 a	442.340 a	118.440 ab	2251.460 a	1703.667 a	223.217 a	79.199 b	76.215 a	75.550 ab
Pr > F(Model)	0.019	0.003	0.013	<0.0001	<0.0001	0.011	0.004	0.029	0.000	<0.0001	0.020	0.031

Table S15. Confusion matrix for the cross-validation dataset.

from \ to	CH	SA	Total	% correct
CH	25	0	25	100.00%
SA	4	20	25	82.61%
Total	29	20	49	91.30%

Table S16. confusion matrix for the validation samples.

from \ to	CH	SA	Total	% correct
CH	1	0	1	100.00%
SA	0	2	2	100.00%
Total	1	2	3	100.00%

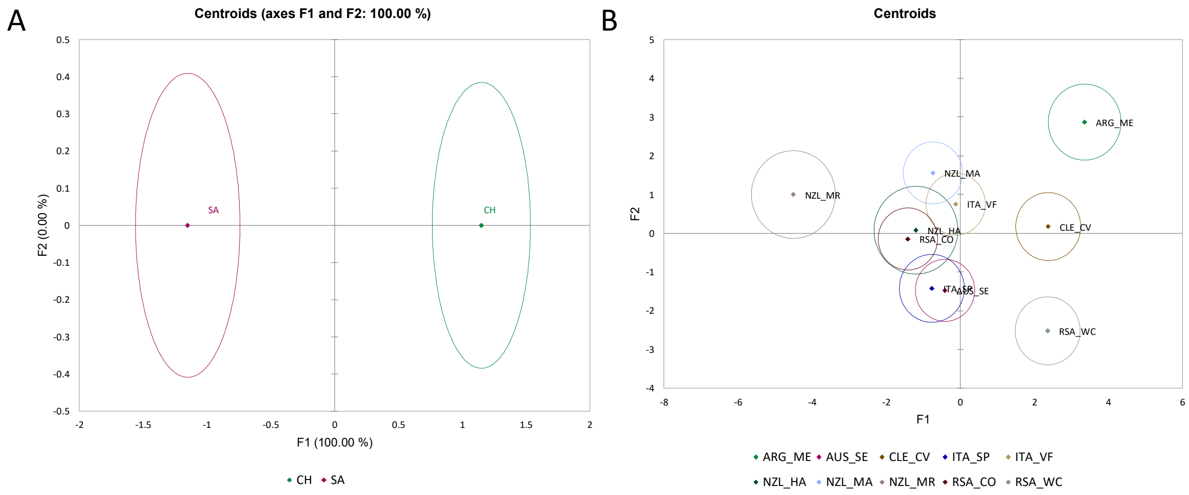

Figure S8. (A) canonical space plots for classification of the varieties and (B) for the classification of the origin.

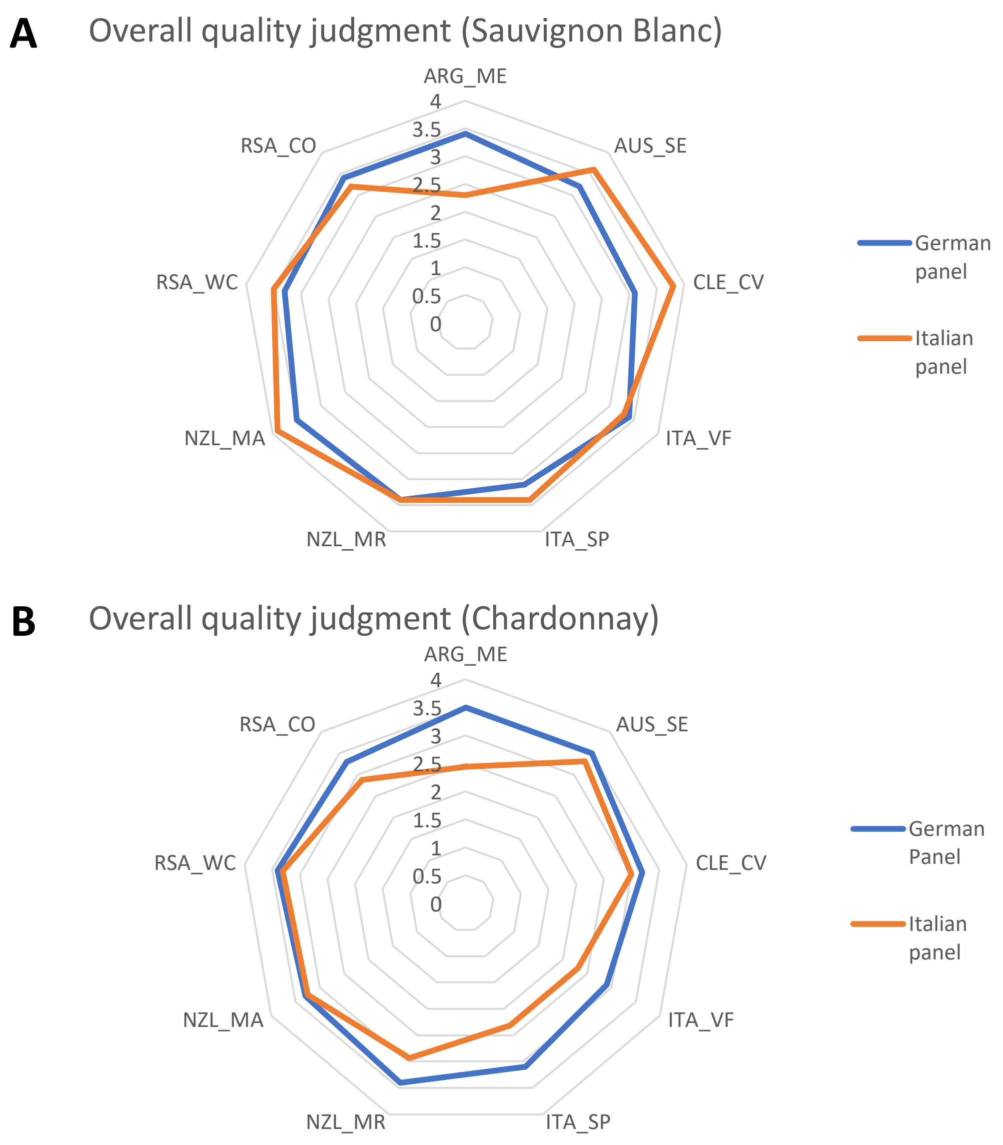

Figure S9. Spider plots for the overall quality judgment comparison between German panel and Italian panel; (A) Sauvignon Blanc OQJ data and (B) Chardonnay OQJ data.
